# Bibliometric and Visual Analysis of Research on the Links Between the Gut Microbiota and Depression From 1999 to 2019

**DOI:** 10.3389/fpsyt.2020.587670

**Published:** 2021-01-08

**Authors:** Xiuqing Zhu, Jinqing Hu, Shuhua Deng, Yaqian Tan, Chang Qiu, Ming Zhang, Xiaojia Ni, Haoyang Lu, Zhanzhang Wang, Lu Li, Hongzhen Chen, Shanqing Huang, Tao Xiao, Dewei Shang, Yuguan Wen

**Affiliations:** ^1^Department of Pharmacy, The Affiliated Brain Hospital of Guangzhou Medical University (Guangzhou Huiai Hospital), Guangzhou, China; ^2^Guangdong Engineering Technology Research Center for Translational Medicine of Mental Disorders, Guangzhou, China

**Keywords:** gut microbiota, depression, bibliometric analysis, citespace, developing trends, microbiota-gut-brain axis, cytokines, microbiome

## Abstract

**Background:** There is a crucial link between the gut microbiota and the host central nervous system, and the communication between them occurs *via* a bidirectional pathway termed the “microbiota-gut-brain axis.” The gut microbiome in the modern environment has markedly changed in response to environmental factors. These changes may affect a broad range of host psychiatric disorders, such as depression, by interacting with the host through metabolic, immune, neural, and endocrine pathways. Nevertheless, the general aspects of the links between the gut microbiota and depression have not been systematically investigated through bibliometric analysis.

**Aim:** This study aimed to analyze the current status and developing trends in gut microbiota research in the depression field through bibliometric and visual analysis.

**Methods:** A total of 1,962 publications published between 1999 and 2019 were retrieved from the Web of Science Core Collection. CiteSpace (5.6 R5) was used to perform collaboration network analysis, co-citation analysis, co-occurrence analysis, and citation burst detection.

**Results:** The number of publications has been rapidly growing since 2010. The collaboration network analysis revealed that the USA, University College Cork, and John F. Cryan were the most influential country, institute, and scholar, respectively. The most productive and co-cited journals were *Brain Behavior and Immunity* and *Proceedings of the National Academy of Sciences of the United States of America*, respectively. The co-citation analysis of references revealed that the most recent research focus was in the largest theme cluster, “cytokines,” thus reflecting the important research foundation in this field. The co-occurrence analysis of keywords revealed that “fecal microbiota” and “microbiome” have become the top two research hotspots since 2013. The citation burst detection for keywords identified several keywords, including “Parkinson's disease,” “microbiota-gut-brain axis,” “microbiome,” “dysbiosis,” “bipolar disorder,” “impact,” “C reactive protein,” and “immune system,” as new research frontiers, which have currently ongoing bursts.

**Conclusions:** These results provide an instructive perspective on the current research and future directions in the study of the links between the gut microbiota and depression, which may help researchers choose suitable cooperators or journals, and promote their research illustrating the underlying molecular mechanisms of depression, including its etiology, prevention, and treatment.

## Introduction

**Figure 1:**
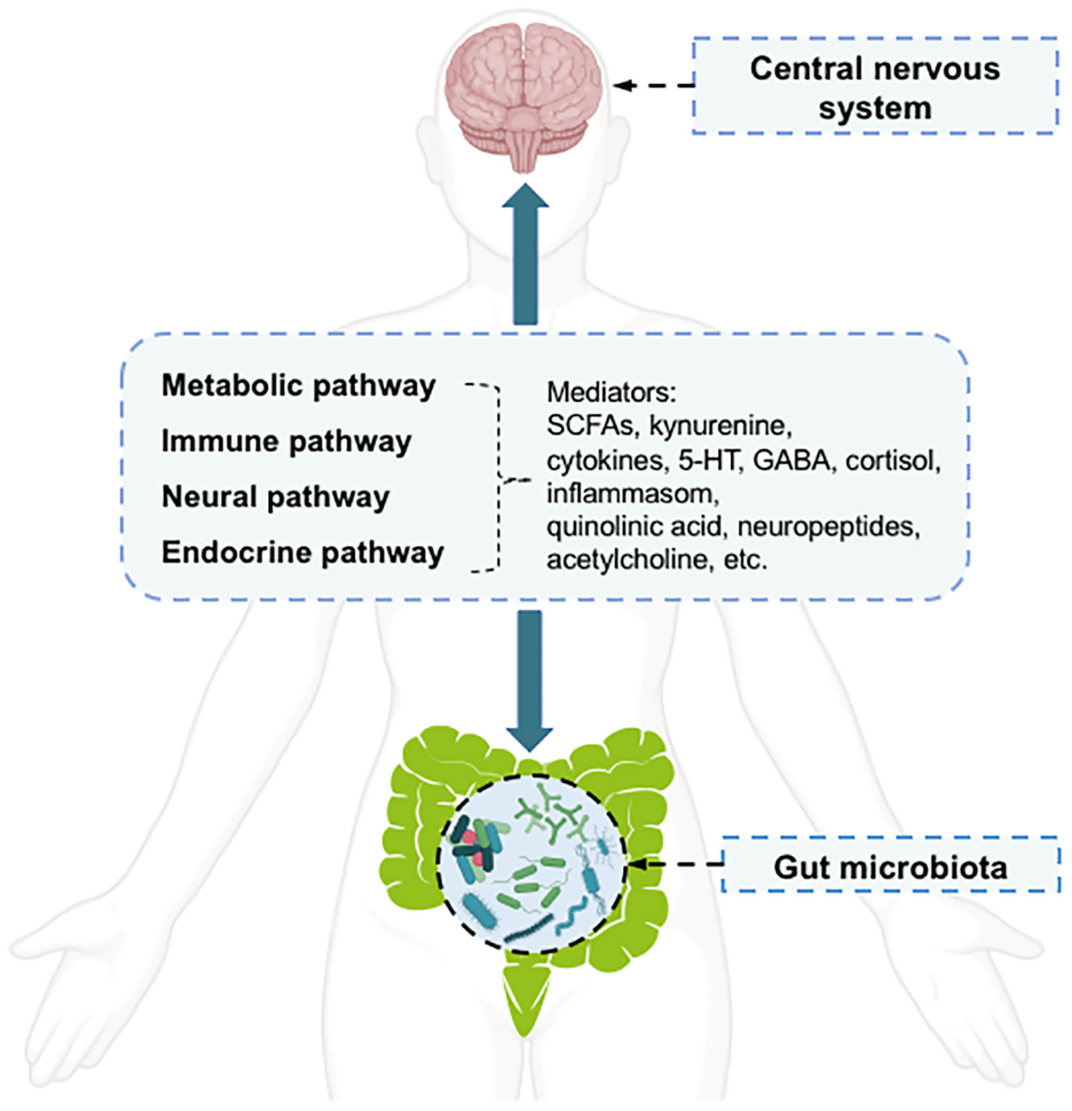
The pathways and mediators of gut microbiota-central nervous system bidirectional communication.

Depression is a common mental illness that can affect both mental and physical health (1). It affects ~350 million of the world's population, as reflected in limited functioning and diminished quality of life (2). According to the Global Burden of Disease Study, in 2017, depression disorders ranked as the third leading cause of global years lived with disability in females, and the fifth leading cause in males (3). However, the pathophysiology of depression disorders has not been fully elucidated, and no single mechanism can adequately explain all aspects of this disease (4). The key molecular mechanisms related to depression disorders include “the monoamine hypothesis,” “hypothalamic-pituitary-adrenal (HPA) axis changes,” “inflammation,” “neuroplasticity and neurogenesis,” “structural and functional brain changes,” “genes,” “environmental milieu,” and “epigenetics (gene-environment interactions)” (4). Therefore, multiple biological and psychosocial determinants are known to be important factors influencing depression (4). Among the factors associated with depression pathology, the gut microbiota can affect a broad range of host psychiatric disorders, such as depression, by interacting with the host through metabolic, immune, neural, and endocrine pathways (5, 6). The communication between the host central nervous system (CNS) and the human gut microbiota occurs *via* a bidirectional pathway, termed the “microbiota-gut-brain axis,” in which the microbiota and its metabolism are major components (7, 8). The communication pathways linking the gut microbiota with the CNS are incompletely understood but appear to have four main routes (6) (Figure 1). Microbial metabolism affects mediators of gut-brain-communication, including neurotransmitters [e.g., serotonin (5-HT) and γ-aminobutyric acid (GABA)], short-chain fatty acids (SCFAs) (e.g., butyrate), hormones (e.g., cortisol), and immune system modulators (e.g., quinolinic acid) (5). The metabolites modulated by the gut microbiota can influence mood, possibly through the activation of peripheral receptors on immune, neural, or endocrine pathways (8). In addition to the peripheral stimulation of these mediators, other direct and indirect mechanisms have been proposed to explain how metabolites affect depressive behavior, such as through direct stimulation of central receptors and epigenetic regulation of histone acetylation or DNA methylation (8).

Compared with our ancestral microbiome, the gut microbiome in the modern environment has been markedly altered because of some environmental factors, such as diet, antibiotic exposure, and proton pump inhibitors. These differences may have important effects on host brain health (9). Fortunately, the rapid development of culture-independent molecular methods (e.g., 16S ribosomal RNA and metagenomic sequencing analysis) (10), metabolomics (11), available animal models of depression (12), and modern neuroimaging and computational biology (13, 14), has enabled quantitative analysis of the components of the gut microbiome and microbial metabolism, and exploration of the pathophysiological roles of the gut microbiota in depression and microbiome-gut-brain interactions. All aspects of these techniques are contributing to gut microbiota research, thus making the gut microbiota a hotspot in depression research. In recent decades, the relationships between the gut microbiota and depression in animals and humans have been widely studied (6). For instance, many probiotic strains known as “psychobiotics,” such as *Lactobacillus paracasei* PS23 and *Lactobacillus helveticus* NS8 (15, 16), as well as multi-species probiotics (17), have been shown to have antidepressant-like effects in rodent studies. Multiple studies in animal models have presented evidence that depression leads to microbiome changes or microbiota changes lead to depression, and indicated a causal relationship between them (6). Many human studies have also shown that probiotics are associated with a significant reduction in depression (18), possibly through an anti-inflammatory mechanism (19). Additionally, some clinical trials have been conducted to explore the feasibility of probiotics as adjuvants for traditional antidepressant therapies (20, 21). Although research on the connection between the gut microbiota and depression is widely available worldwide, the general aspects of this Research Topic, to our knowledge, have not been systematically analyzed through bibliometric analysis. Therefore, our study aimed to fill this gap in the literature.

Bibliometrics involves the use of a series of defined metrics and enables researchers to assess the published research output, impact, and developing trends by using statistical and quantitative methods (22). It can allow researchers to extract quantitative information on distribution by country/region, institution, author, and journal, and can aid in identifying research hotspots and frontiers in a particular field in a short time. Over the past 10 years, bibliometrics has been widely used to analyze scientific research in medical research worldwide (23). Previous bibliometric studies have focused on the gastrointestinal microbiome (24), the intestinal microbiota in obesity (25), or the microbiome-gut-brain axis (26), but have not addressed the gut microbiota in depression. Our bibliometric analysis involves this new and important field. The results should be helpful for researchers examining the gut microbiota in the depression field, aiding them in identifying journals to publish in and collaborators. The timely review and analysis of the hotspots and research trends may also promote the development of this field and advance research illustrating the molecular mechanisms underlying depression, such as its etiology, prevention, and treatment.

Therefore, this study aimed to comprehensively analyze the current status and developing trends in publications between 1999 and 2019 in gut microbiota research in the depression field through a bibliometric and visual analysis. The Web of Science™ Core Collection (WoSCC) database and CiteSpace software were used to conduct the bibliometric and visual analysis. Our focus was to: (i) investigate the outputs and growth trends in publications; (ii) construct international scientific collaboration networks among countries/regions, institutions, and authors; (iii) determine the core countries/regions, institutions, researchers, and journals; and (iv) explore the key topics, hotspots, and research trends, to guide future research and applications.

## Materials and Methods

### Introduction to CiteSpace

Science mapping, one of the main approaches used to explore a field of research in bibliometrics, is a general process of domain analysis and visualization. Its research scope includes a scientific discipline, and a research field or topics of particular research questions (27). CiteSpace, the Java application chosen to perform the bibliometric analysis, was developed in 2004 by Professor Chaomei Chen (College of Computing and Informatics, Drexel University, Philadelphia, PA, USA), an international expert in the information visualization field (28). It is an interactive analytic tool enabling visualization tasks in science mapping through a combination of bibliometrics, visual analytic methods, and data mining algorithms. CiteSpace supports several types of bibliometric studies, including collaboration network analysis, co-citation analysis, and co-occurrence analysis, and can generate visual maps such as geospatial visualizations (27). Collaboration network analysis can identify the core countries/regions, institutions, and authors in a particular field as well as their cooperative relationships. Additionally, co-citation analysis and co-occurrence analysis can reflect the research foundation and hotspots of the field, respectively. To date, it has been continually developed and widely used in the field of medical research (29, 30).

### Data Acquisition and Search Terms

Bibliometric analysis relies on literature databases. One of the best-known databases is the WoSCC database, a curated collection of high-quality scholarly content on the Web of Science™ platform (WoS; previously known as Web of Knowledge), an online subscription-based scientific citation indexing service maintained by Thomson Reuters (31). To avoid the bias due to the daily database updates, because the database remains open, we performed the literature retrieval from WoSCC on a single day, May 15, 2020. The search index used in WoSCC was standardized to include all the relevant publications to ensure the comprehensiveness of the bibliographic data.

Synonyms for gut microbiota and depression were included in the search strategy, as follows: TS = (((gut OR intestin^*^ OR gastrointestin^*^ OR gastro-intestin^*^) AND (microbiot^*^ OR microbiome^*^ OR flora OR microflora OR bacteria)) OR prebiotic OR probiotic OR antibiotic OR dysbiosis) AND TS = (depression OR depressed OR depressions OR depressive OR despondent OR gloomy). In the present study, the inclusive and exclusion criteria were as follows: (i) the timespan ranged from 1999 to 2019, encompassing 20 years in total, (ii) only articles and reviews were included, whereas other document types (e.g., letters, meeting abstracts, retracted publications, and book chapters) were excluded, (ii) no species limitations were set, (iv) the publication language was restricted to English, and (v) duplicate publications were excluded. The search results were directly analyzed with the WoSCC literature analysis wire and exported for further analysis in CiteSpace. The present study used published data from secondary sources and did not involve any interactions with human subjects; hence, the requirement for institutional review board approval was waived (32).

### Analysis Tools

Features such as the publication outputs, subject categories of WoS, *h*-index, and impact factor (IF) were analyzed with the WoSCC literature analysis wire (33). The *h*-index is a measure of productivity and the impact of a researcher who has published *h* articles cited at least h times each; that is, if the *h*-index of a scientist is 10, the scientist published 10 articles with at least 10 citations per article (34). The IF from Thomson Reuters indicates the impact of journals, referring to the number of citations to a given journal in a specific year (35).

CiteSpace (5.6 R5) (http://cluster.cis.drexel.edu/~cchen/citespace/) was used to perform collaboration network analysis (authors, countries/regions, and institutions), co-citation analysis (journals, authors, and references), analysis of keyword co-occurrence, and citation burst detection for keywords and references. The specific parameters used in CiteSpace were set as follows: time slicing (from 1999 to 2019, years per slice = 1), term source (title, abstract, author, keyword, and keywords plus), node type (one option chosen at a time from author, institution, country, keyword, cited reference, cited author, and cited journal), link strength (cosine), link scope (within slices), selection criteria (top 50 per slice), and pruning (none).

The visualizations generated by CiteSpace consisted of nodes and link lines. The nodes in the network maps represented the type of study being analyzed, such as the authors, countries/regions, institutions, cited references, and keywords; link lines between the nodes indicated cooperative, co-cited, or co-occurring relationships (36). The size of a node indicated the number of citation or occurrence, and the width of a line indicated the strength of the relationship (36, 37). The color of a node, represented by a series of annual rings, indicated the distribution time (36, 38). The importance of the publication was identified and we measured its betweenness centrality. A key node or pivot point in the scientific collaboration or reference co-citation network was marked with a purple ring if its centrality was ≥ 0.1 (39, 40). The calculated Q-value and silhouette-value were indicators representing the modularity and homogeneity of the cluster network, respectively. The larger the Q-value, the better the modularity. The closer the silhouette-value to zero, the higher the homogeneity. A Q-value > 0.3 identified the cluster structure as significant, and a mean silhouette > 0.5 or > 0.7 indicated that the clustering result was reasonable or highly credible, respectively (40). A citation burst had two attributes: the intensity and the duration of the burst. The burst detection revealed abrupt changes in terms or citations over a specified period, thereby identifying emerging research trends (41).

IBM SPSS Statistics, version 25.0 (SPSS Inc., Chicago, IL, USA) was used to depict and visualize the growth trend in annual publication outputs by using a polynomial regression model. We tested for possible correlation relationships between the total number of publication outputs and the betweenness centrality for each country/region, institution, and author that reached a centrality threshold above zero. Pearson's correlation coefficient (*r*) was calculated in SPSS. A *p*-value < 0.05 was considered to indicate statistical significance.

## Results

### Analysis of Publication Outputs and Growth Trend Prediction

**Figure 2:**
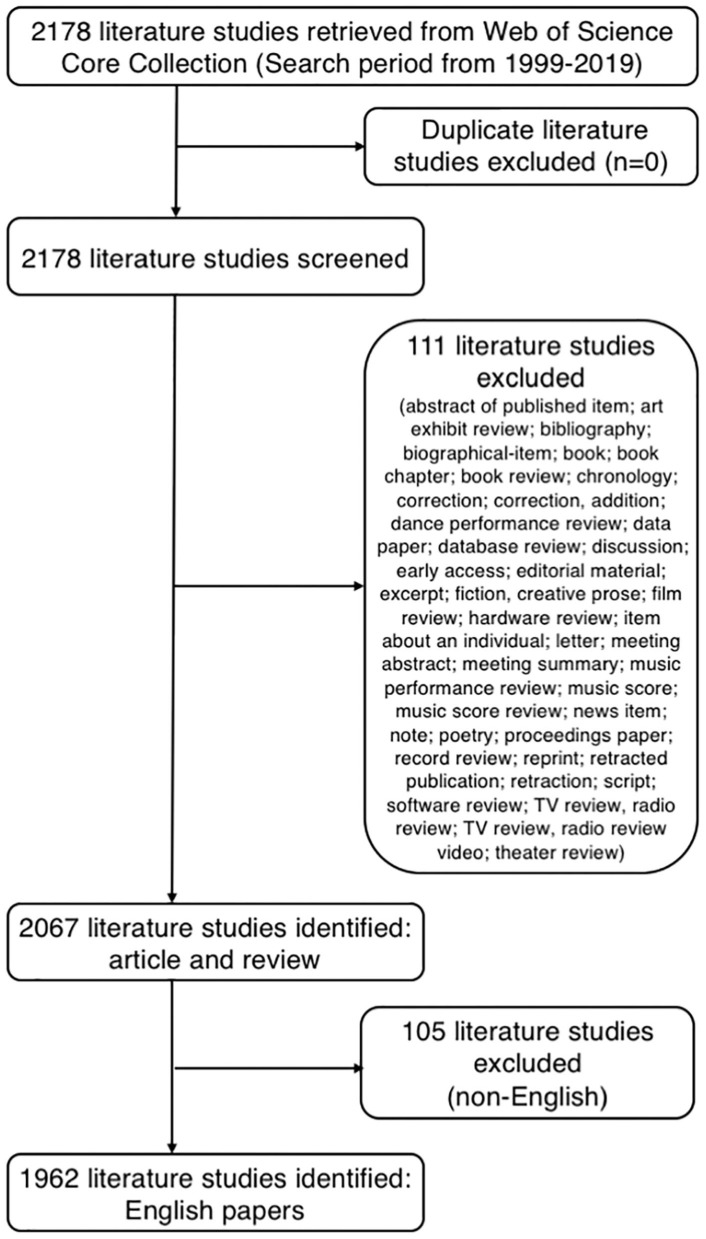
Flowchart for including and excluding literature studies.

**Figure 3:**
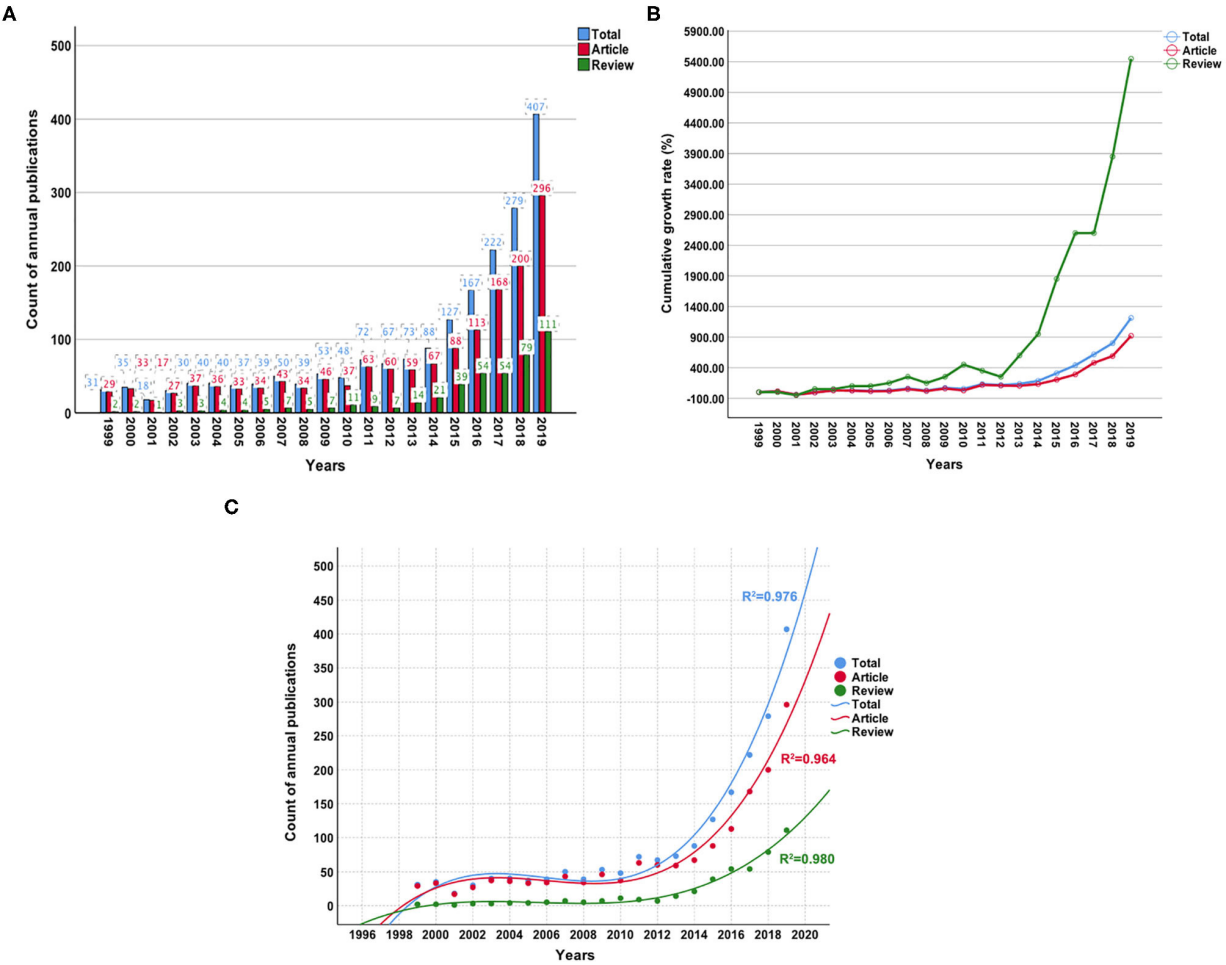
Trends in the number of publications from 1999 to 2019 on gut microbiota research in the depression field. **(A)** The annual number of publications. **(B)** The cumulative growth rate for publications. **(C)** The polynomial fitting curves of growth trends.

The search retrieved 1,962 publications that met the inclusion and exclusion criteria (Figure 2), and 1,520 and 442 articles and reviews, respectively, were identified. Over the past 20-year period, the development track showed two stages: one was the initial period (1999–2009), which had a very slow development speed, and the other was a rapid development period (2010–2019). The number of publication outputs increased from 31 publications in 1999 to 407 publications in 2019, with an average of 98 publications per year (Figure 3A). Compared with those in the year 1999, the cumulative growth rate reached 1212.903, 920.690, and 5450.000% in 2019 for total publications, articles, and reviews, respectively, and the compound annual growth rate (CAGR) was 13.740, 12.317, and 22.240%, respectively (Figure 3B). The CAGR was the annualized average rate of growth between the years 1999 and 2019, calculated as follows: CAGR = [(value in year 2019/value in year 1999) ^(1/20)^-1]. As presented in Figure 3C, the publication trend remained relatively limited and stable before the 2010s, whereas continual growth of research on gut microbiota in the depression field has occurred since then. The model fitting curves of growth in document number showed a strongly positive correlation with the year of publication (*R*^2^ = 0.976, 0.964, and 0.980 for total publications, articles, and reviews, respectively). We conservatively estimated that the total publication, article, and review outputs will exceed 450, 325, and 125, respectively, in 2020. The top five WoS categories of the analyzed publications were neurosciences (*n* = 322, 16.412%), psychiatry (*n* = 240, 12.232%), pharmacology/pharmacy (*n* = 194, 9.888%), clinical neurology (*n* = 123, 6.269%), and gastroenterology/hepatology (*n* = 119, 6.065%).

### Analysis of Scientific Collaboration Network

**Figure 4:**
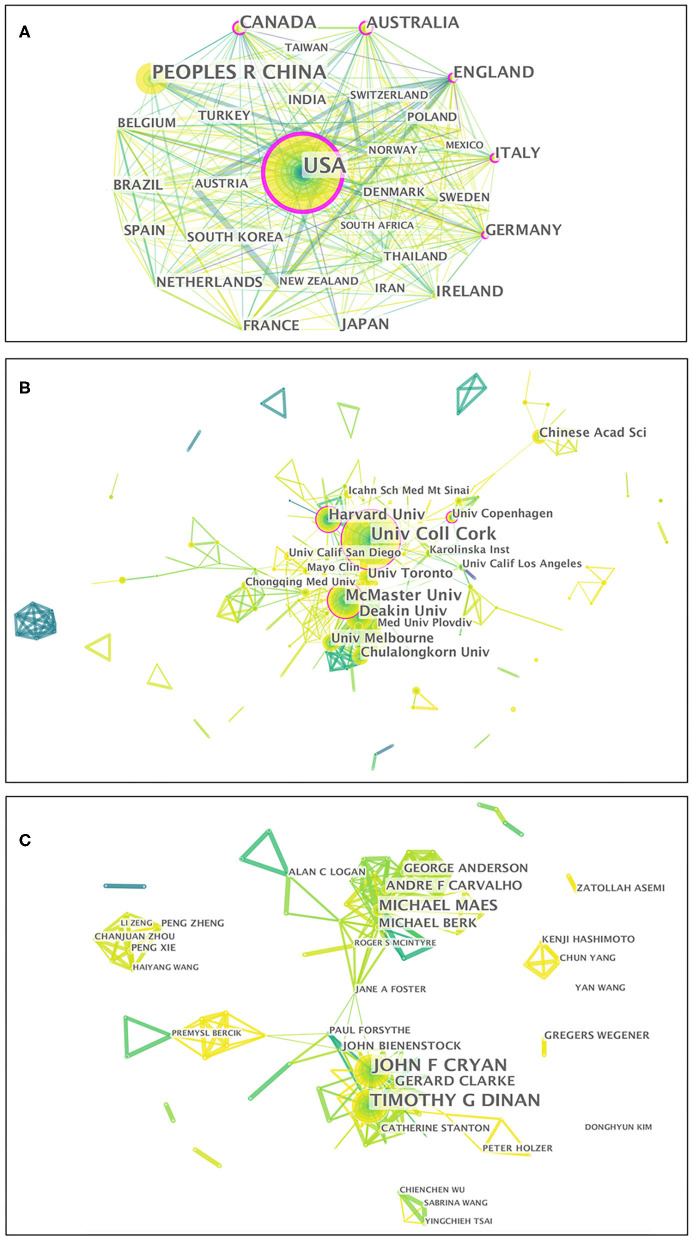
Visualization map of the scientific collaboration network analysis of gut microbiota research in the depression field from 1999 to 2019. Collaboration among countries/regions **(A)**, institutions **(B)**, and authors **(C)**. The nodes in the map denote elements such as an author, country/region, or institute, and link lines between nodes denote collaborative relationships. The larger the circle, the more articles published. The wider the line, the stronger the relationship. The outermost purple ring denotes the centrality level, and highly central nodes are considered pivotal points in the research field.

Fifty-two countries/regions contributed to the publications on gut microbiota research in the depression field. The top 10 countries/regions according to publications and centrality are listed in Table 1. The top five countries/regions, in order of the number of publications, were the USA, People's Republic of China, Canada, Australia, and England. The top five countries/regions by centrality were the USA, Germany, Australia, England, and Italy. Pearson's correlation analysis revealed a significant correlation between publications and centrality at the country/region level (*r* = 0.861, *p* < 0.001). Comprehensive analyses of publications and centrality indicated that the USA (publications: 580, centrality: 0.35), Australia (publications: 120, centrality: 0.16), and England (publications: 118, centrality: 0.16) were the most influential in the field. The collaboration network map among countries/regions is presented in Figure 4A, in which there were 52 nodes and 319 link lines. The nodes and the link lines between them represent the countries/regions and their cooperative relationships, respectively. The larger the node, the more publications. The wider the line, the stronger the relationships. Changes in node color represent the distribution time of publications. The node marked with a purple ring represents centrality ≥ 0.1. Overall, the USA (collaborators: 38), England (collaborators: 29), Germany (collaborators: 29), Canada (collaborators: 28), and Australia (collaborators: 28) had the largest number of national partners.

**Table 1:** The top 10 countries/regions, institutions, and authors in terms of publications and centrality.

**Items**	**Publications**			**Centrality**		
	**Ranking**	**Name**	**Number**	**Ranking**	**Name**	**Number**
Country/Region	1	USA	580	1	USA	0.35
	2	Peoples R China	257	2	Germany	0.17
	3	Canada	158	3	Australia	0.16
	4	Australia	120	4	England	0.16
	5	England	118	5	Italy	0.12
	6	Italy	89	6	Canada	0.11
	7	Germany	86	7	Peoples R China	0.10
	8	Ireland	84	8	Belgium	0.07
	9	Japan	84	9	Brazil	0.05
	10	France	61	10	Ireland	0.04
Institution	1	University College Cork	74	1	University College Cork	0.19
	2	McMaster University	47	2	McMaster University	0.16
	3	Deakin University	37	3	Catholic University of Louvain	0.13
	4	Harvard University	34	4	University of Copenhagen	0.12
	5	University of Toronto	24	5	Harvard University	0.11
	6	University of Melbourne	24	6	University of Toronto	0.09
	7	Chulalongkorn University	23	7	University of Queensland	0.08
	8	Chinese Academy of Sciences	20	8	University of Auckland	0.08
	9	University of Copenhagen	15	9	University of Adelaide	0.08
	10	University of California, San Diego	13	10	Jiangnan University	0.08
Author	1	John F. Cryan	60	1	John F. Cryan	0.03
	2	Timothy G Dinan	57	2	Jane A Foster	0.03
	3	Michael Maes	30	3	Michael Maes	0.02
	4	Gerard Clarke	20	4	Timothy G Dinan	0.01
	5	Michael Berk	16	5	Michael Berk	0.01
	6	André F Carvalho	15	6	André F Carvalho	0.01
	7	George Anderson	13	7	John Bienenstock	0.01
	8	John Bienenstock	12	8	Paul Forsythe	0.01
	9	Gregers Wegener	9	9	Michael G Surette	0.01
	10	Catherine Stanton	8	10	Felice N Jacka	0.01

From 1999 to 2019, a total of 266 institutions published in this field. Table 1 illustrates the top ten institutions according to publications and centrality. The top five institutions with the greatest contribution to this field were University College Cork, McMaster University, Deakin University, Harvard University, and University of Toronto. In terms of centrality, the top five institutions were University College Cork, McMaster University, Catholic University of Louvain, University of Copenhagen, and Harvard University. A significant correlation between publications and centrality was also observed at the institution level (Pearson *r* = 0.734, *p* < 0.001). On the basis of the analyses of publications and centrality, University College Cork (publications: 74, centrality: 0.19), McMaster University (publications: 47, centrality: 0.16), and Harvard University (publications: 34, centrality: 0.11) were the major research institutions. The generated institution network map identified 266 nodes and 581 link lines, which represented the institutions and their cooperative relationships, respectively; thus extensive collaborations between institutions were found (Figure 4B). For instance, University College Cork, represented by the largest node marked with a purple ring, had the most publications and the most extensive cooperation, with more than 25 institutions, such as TEAGASC, McMaster University, Harvard University, Deakin University, University of Auckland, University of Gothenburg, University of California-Los Angeles, and Catholic University of Louvain. Although Catholic University of Louvain did not have as many publications, it had many research partners, such as Medical University of Graz, University of Reading, University College Cork, McMaster University, University of Copenhagen, and University of Auckland.

In total, 368 authors published in this field in the past two decades. Table 1 presents the top 10 authors according to publications and centrality. Eight authors published more than 10 publications in this field. Among these active authors, John F. Cryan was ranked first, with 60 publications, followed by Timothy G. Dinan and Michael Maes. The top three authors ranked by centrality were John F. Cryan, Jane A. Foster, and Michael Maes, whereas none had a centrality ≥ 0.1, thus indicating that international cooperation among top scientific researchers was insufficient. Furthermore, there was no significant correlation between publications and centrality at the author level (Pearson *r* = 0.380, *p* = 0.249). The co-authorship network map is shown in Figure 4C, containing 368 nodes and 924 collaboration lines. The nodes and the link lines between them represent the authors and their cooperative relationships, respectively. None of the nodes are marked with a purple ring, owing to their very low centrality (<0.1). As presented in the map, Michael Maes, represented by the third largest node, had restricted relationships with John F. Cryan or Timothy G. Dinan. Notably, Jane A. Foster played a central role in international collaborations but did not have considerable publication productivity.

### Analysis of Journals and Co-cited Journals

The articles included were published in 1,038 different journals, many of which were specialized journals. As one of the most important indicators, co-citation analysis has been widely used in bibliometrics. Table 2 presents the top 10 journals and co-cited journals for gut microbiota research in the depression field. The most prolific journal in this field was *Brain Behavior and Immunity*, followed by *PLoS One, Poultry Science*, and *Scientific Reports*. All the highly productive journals had an IF above 2.0. Co-cited journals were those cited together by other researchers. The top three journals according to co-citation count were *Proceedings of the National Academy of Sciences of the United States of America* (*PNAS*), *PLoS One*, and *Nature*. The journals with betweenness centrality ≥ 0.1 included *PNAS* (centrality: 0.20), *British Journal of Nutrition* (citations: 432, centrality: 0.16), *Science* (centrality: 0.12), and *Nature* (centrality: 0.11). The published references of those top journals reflect the foundation of this field.

**Table 2:** The top 10 journals and co-cited journals for gut microbiota research in the depression field.

**Items**	**Ranking**	**Name**	**Country**	**Counts**	**IF (2019)**
Journal	1	*Brain Behavior and Immunity*	USA	34	6.633
	2	*Plos One*	USA	34	2.740
	3	*Poultry Science*	USA	23	2.659
	4	*Scientific Reports*	England	21	3.998
	5	*Journal of Affective Disorders*	Netherlands	16	3.892
	6	*Nutrients*	Switzerland	15	4.546
	7	*Neurogastroenterology and Motility*	England	14	3.008
	8	*Translational Psychiatry*	USA	14	5.280
	9	*Frontiers in Psychiatry*	USA	13	2.849
	10	*Journal of Animal Science*	USA	13	2.092
Co-cited Journal	1	*Proceedings of the National Academy of Sciences of the United States of America*	USA	797	9.412
	2	*Plos One*	USA	786	2.740
	3	*Nature*	England	683	42.778
	4	*Gastroenterology*	USA	623	17.373
	5	*Brain Behavior and Immunity*	USA	603	6.633
	6	*Science*	USA	566	41.845
	7	*Biological Psychiatry*	USA	566	12.095
	8	*Neurogastroenterology and Motility*	England	547	3.008
	9	*Gut*	England	532	19.819
	10	*Molecular Psychiatry*	England	464	12.384

### Analysis of Co-cited Authors

Co-cited authors are those cited together by other researchers. The co-citation analysis by author included 678 cited authors, 14 of whom were cited at least 200 times. The top 10 co-cited authors and their affiliates, major research fields, and *h*-indexes are listed in Table 3. The top three co-cited authors all came from Ireland. Lieve Desbonnet ranked first, with 315 citations, followed by Timothy G. Dinan, John F. Cryan, and Javier A. Bravo, whereas the remaining authors had fewer than 300 citations. The uppermost *h*-index value was for Michael Maes, followed by John F. Cryan and Timothy G. Dinan. Their research areas were mainly neurosciences, psychiatry, and pharmacology. These authors contributed to the work in this field, which provided the research basis.

**Table 3:** The top 10 co-cited authors with the most citations.

**Ranking**	**Times cited**	**Author**	**Institution (Country)**	**Major research fields**	***h*-index**
1	315	Lieve Desbonnet	National University of Ireland Galway/ Royal College of Surgeons (Ireland)	Neurosciences/Neurology, Psychiatry, Pharmacology/Pharmacy	16
2	314	Timothy G. Dinan	University College Cork (Ireland)	Neurosciences/Neurology, Psychiatry, Pharmacology/Pharmacy	93
3	312	John F. Cryan	University College Cork (Ireland)	Neurosciences/Neurology, Pharmacology/Pharmacy, Psychiatry	95
4	301	Javier A. Bravo	Pontificia Universidad Católica de Valparaíso (Chile)	Neurosciences/Neurology, Pharmacology/Pharmacy, Gastroenterology/Hepatology	19
5	276	Premysl Bercik	McMaster University (Canada)	Gastroenterology/Hepatology, Neurosciences/Neurology, Microbiology	43
6	260	Nobuyuki Sudo	Kyushu University (Japan)	Neurosciences/Neurology, Endocrinology Metabolism, General Internal Medicine	29
7	260	Michael Maes	Chulalongkorn University (Thailand)	Neurosciences/Neurology, Psychiatry, Pharmacology/Pharmacy	95
8	255	Michael Messaoudi	ETAP Appl Ethol (France)	Nutrition/Dietetics, Pharmacology/Pharmacy, Biochemistry Molecular Biology	19
9	222	Siobhain M. O'Mahony	University College Cork (Ireland)	Neurosciences/Neurology, Psychiatry, Pharmacology/Pharmacy	26
10	207	John R. Kelly	Trinity College Dublin (Ireland)	Psychiatry, Neurosciences/Neurology, Pharmacology/Pharmacy	9

### Analysis of Co-cited References

**Figure 5:**
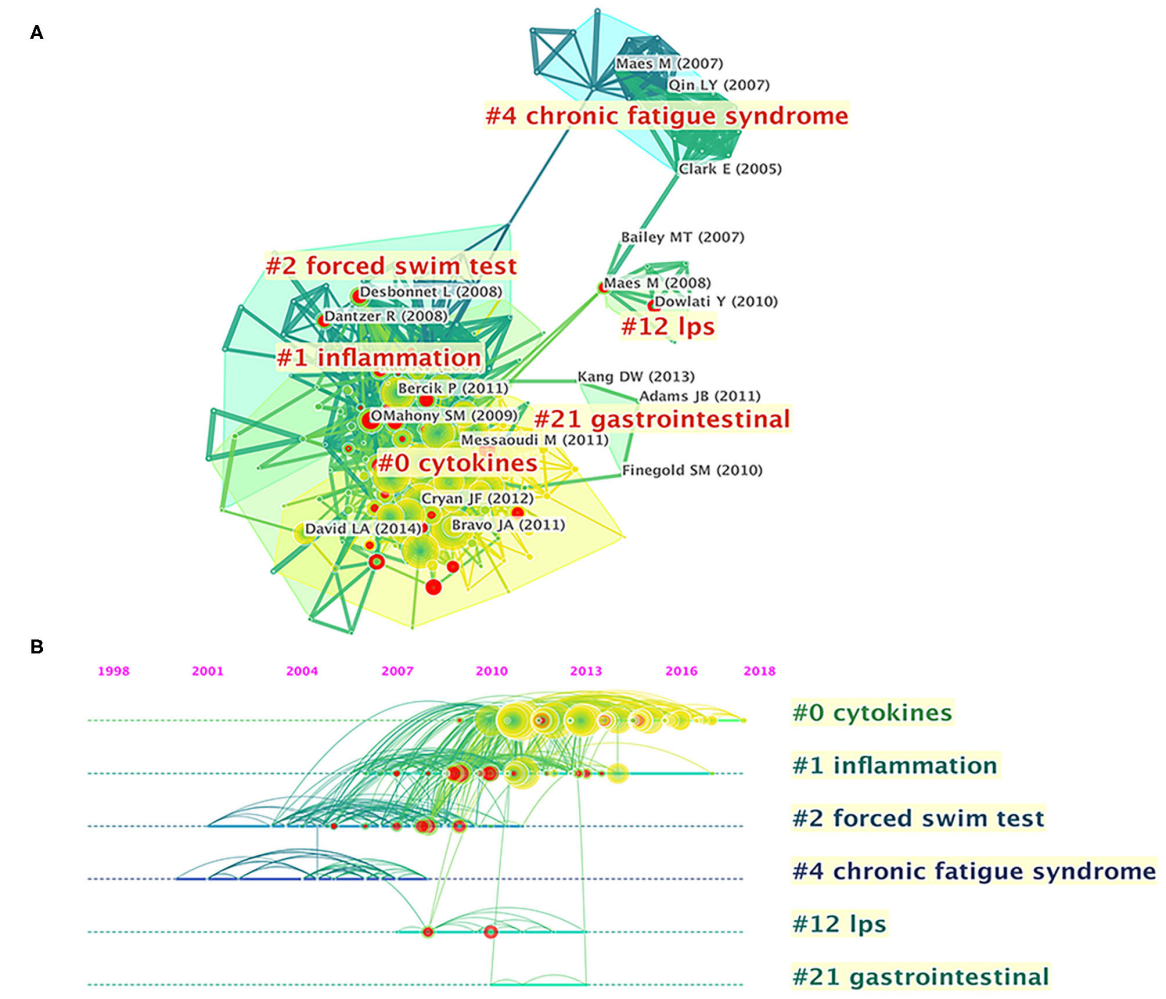
Reference co-citation network analysis of publications on gut microbiota research in the depression field between 1999 and 2019. **(A)** Cluster visualization of the reference co-citation map. **(B)** Timeline visualization of the reference co-citation map. Top clusters and respective landmark references are labeled. The nodes in the map denote co-cited references, and links between nodes denote co-citation relationships. The citation rings denote the citation history of a reference. Large nodes or nodes with red tree-rings are either highly cited or have citation bursts in a given time slice.

References cited are often considered a core component of bibliometric research. Co-cited references are those co-cited in the reference lists of other articles. Figure 5A shows a cluster visualization of the reference co-citation network generated by CiteSpace software, which was divided into 90 clusters, of which only the largest six extracted from the references, on the basis of indexing terms and identified by a log-likelihood ratio algorithm. They are shown with different convex hulls in the figure, including cytokines (cluster #0), inflammation (cluster #1), forced swim test (cluster #2), chronic fatigue syndrome (cluster #4), lipopolysaccharide (LPS) (cluster #12), and gastrointestinal (cluster #21). The different nodes in the map represent cited references, and their authors are labeled in black. The representative authors in the largest six clusters are shown in Figure 5A. The details of the largest six clusters of references in the co-citation network are shown in Table 4. The total Q-value was 0.6901, and each cluster had a mean silhouette above 0.5, indicating that the cluster quality was reasonable. Figure 5B shows the top six clusters in a timeline view, which depicts clusters along with horizontal timelines and has a vertical arrangement according to descending size, thus indicating the scientific relevance of the published articles. Figure 5B and Table 4 show that the most recent research focus was in cluster #0, “cytokines” (mean year 2014). The cluster and timeline visualization of the reference co-citation map contained 410 nodes and 1,796 links, representing the cited references and their co-cited relationships, respectively.

**Table 4:** The largest six clusters of references in the co-citation network.

**Cluster**	**Size**	**Mean silhouette**	**Mean year**	**Label (LLR algorithm)**	**Representative reference**
0	70	0.725	2014	Cytokines	Bravo et al. (42)
1	51	0.658	2010	Inflammation	Bercik et al. (43)
2	40	0.816	2007	Forced swim test	Desbonnet et al. (44)
4	21	0.981	2004	Chronic fatigue syndrome	Maes et al. (45)
12	7	0.989	2010	Lipopolysaccharide (LPS)	Maes et al. (46)
21	3	0.968	2011	Gastrointestinal	Finegold et al. (47)

“*LLR” means log-likelihood ratio*.

The characteristics of the top 10 highly co-cited references concerning gut microbiota research in the depression field are summarized in Table 5. All were found in cluster #0, and the top-ranked reference was published by Javier A Bravo (42), with a co-citation count of 283. The articles with the maximum co-citations are generally important foundational studies in this field.

**Table 5:** The top10 co-cited references related to gut microbiota research in the depression field between 1999 and 2019.

**Ranking**	**Cited by**	**Authors**	**Title**	**Source title**	**Year of publication**	**Type of document**
1	283	Bravo et al. (42)	Ingestion of Lactobacillus strain regulates emotional behavior and central GABA receptor expression in a mouse *via* the vagus nerve.	*Proceedings of the National Academy of Sciences of the United States of America*	2011	Article
2	235	Cryan and Dinan (48)	Mind-altering microorganisms: the impact of the gut microbiota on brain and behavior.	*Nature Reviews Neuroscience*	2012	Review
3	207	Messaoudi et al. (49)	Assessment of psychotropic-like properties of a probiotic formulation (*Lactobacillus helveticus* R0052 and *Bifidobacterium longum* R0175) in rats and human subjects.	*British Journal of Nutrition*	2011	Article
4	200	Diaz Heijtz et al. (50)	Normal gut microbiota modulates brain development and behavior.	*Proceedings of the National Academy of Sciences of the United States of America*	2011	Article
5	196	Jiang et al. (51)	Altered fecal microbiota composition in patients with major depressive disorder.	*Brain Behavior and Immunity*	2015	Article
6	177	Bercik et al. (52)	The intestinal microbiota affect central levels of brain-derived neurotropic factor and behavior in mice.	*Gastroenterology*	2011	Article
7	169	Foster and McVey Neufeld (53)	Gut-brain axis: how the microbiome influences anxiety and depression.	*Trends in Neurosciences*	2013	Review
8	167	Clarke et al. (54)	The microbiome-gut-brain axis during early life regulates the hippocampal serotonergic system in a sex-dependent manner.	*Molecular Psychiatry*	2013	Article
9	164	Neufeld et al. (55)	Reduced anxiety-like behavior and central neurochemical change in germ-free mice.	*Neurogastroenterology and Motility*	2011	Article
10	156	Naseribafrouei et al. (56)	Correlation between the human fecal microbiota and depression.	*Neurogastroenterology and Motility*	2014	Article

### Analysis of Co-occurring Keywords

**Figure 6:**
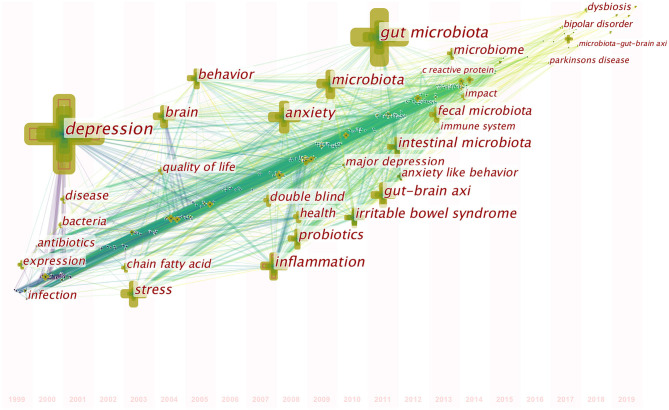
Time-zone view of keyword co-occurrence in publications from 1999 to 2019 on gut microbiota research in the depression field. The nodes represent keywords, and the chronological order in which keywords appear can be found on the map. The size of a node is proportional to the frequency of keyword occurrence.

A map of keyword co-occurrence reflects research hotspots. Figure 6 presents a time-zone view of the keyword co-occurrence network to map the knowledge structure of research, resulting in 350 nodes and 2,168 links, representing the keywords and their co-occurring relationships, respectively. The larger the node, the greater the occurrence the keyword. In terms of co-occurrence frequency, the top 10 keywords were “depression,” “gut microbiota,” “anxiety,” “inflammation,” “microbiota,” “stress,” “irritable bowel syndrome,” “gut-brain axis,” “intestinal microbiota,” and “brain” (Table 6). However, the keywords “fecal microbiota” and “microbiome” have become the top two research hotspots since 2013 (Figure 6), appearing in 98 and 94 citing studies, respectively (Table 6).

**Table 6:** The top 20 keywords in terms of frequency for gut microbiota research in the depression field.

**Ranking**	**Keyword**	**Frequency**	**Ranking**	**Keyword**	**Frequency**
1	Depression	532	11	Probiotics	163
2	Gut microbiota	370	12	Behavior	151
3	Anxiety	225	13	Double blind	103
4	Inflammation	201	14	Fecal microbiota	98
5	Microbiota	195	15	Microbiome	94
6	Stress	185	16	Health	93
7	Irritable bowel syndrome	169	17	Disease	93
8	Gut-brain axis	167	18	Expression	89
9	Intestinal microbiota	167	19	Quality of life	88
10	Brain	164	20	Antibiotics	82

### Analysis of Burst Detection

**Figure 7:**
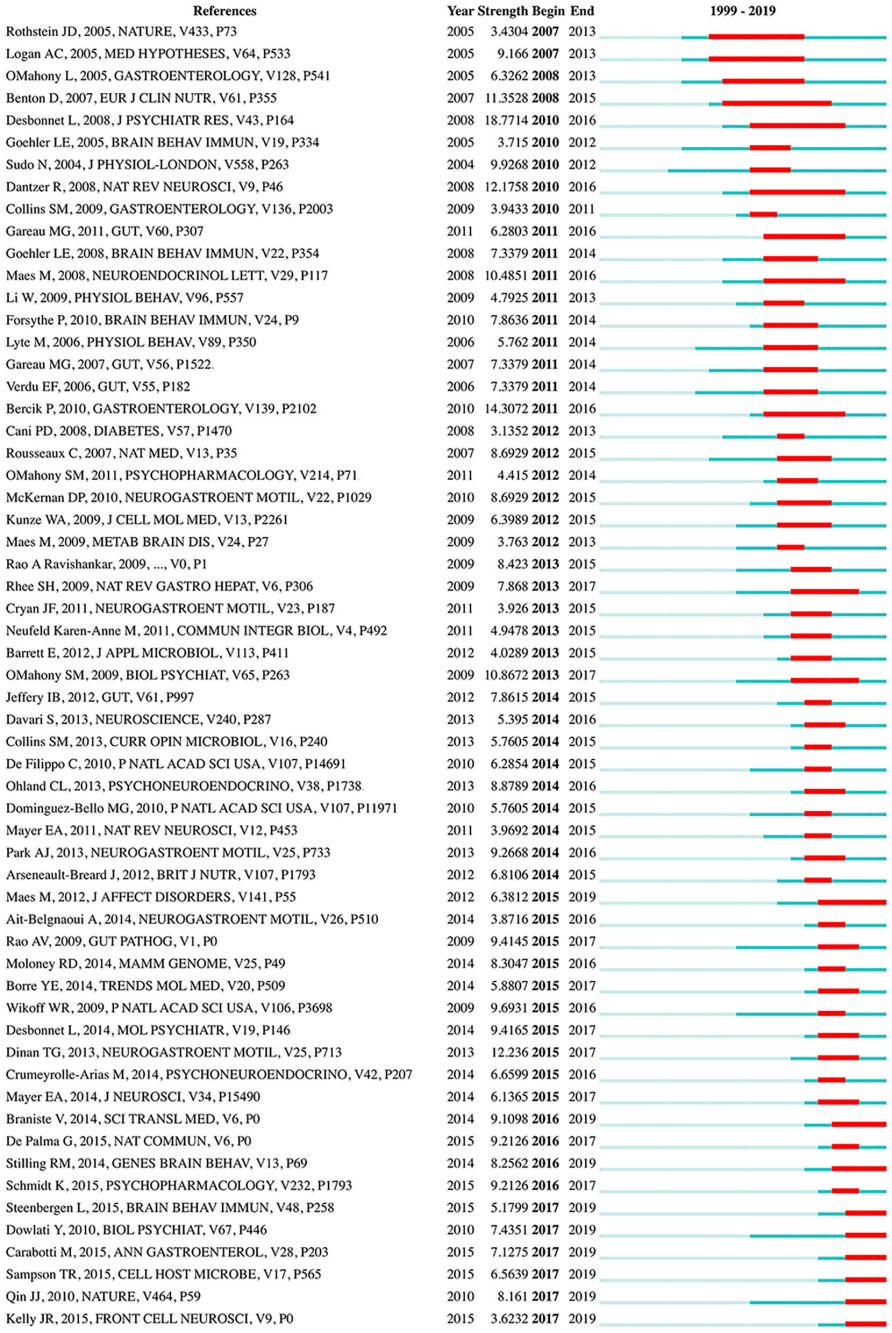
References with the strongest citation bursts in publications from 1999 to 2019 on gut microbiota research in the depression field. The strength values reflect the frequency of citation. The red bars indicate references cited frequently; the green bars indicate references cited infrequently.

References with citation bursts, defined as those that have frequent citations over time, can be used to indicate the evolution of a knowledge domain (57). A total of 59 references with strong citation bursts were detected (Figure 7). The duration of the burst is displayed by a red line segment. References with citation bursts first appeared in 2007 (58, 59), and the most recent references with citation bursts appeared in 2017 (60–65). The strongest burst (strength: 18.7714) appeared in 2010 for a 2008 article (44). Nine references had a burst that lasted until 2019 (60–68). The references with citation bursts between 2010 and 2017 accounted for 93.22%.

**Figure 8:**
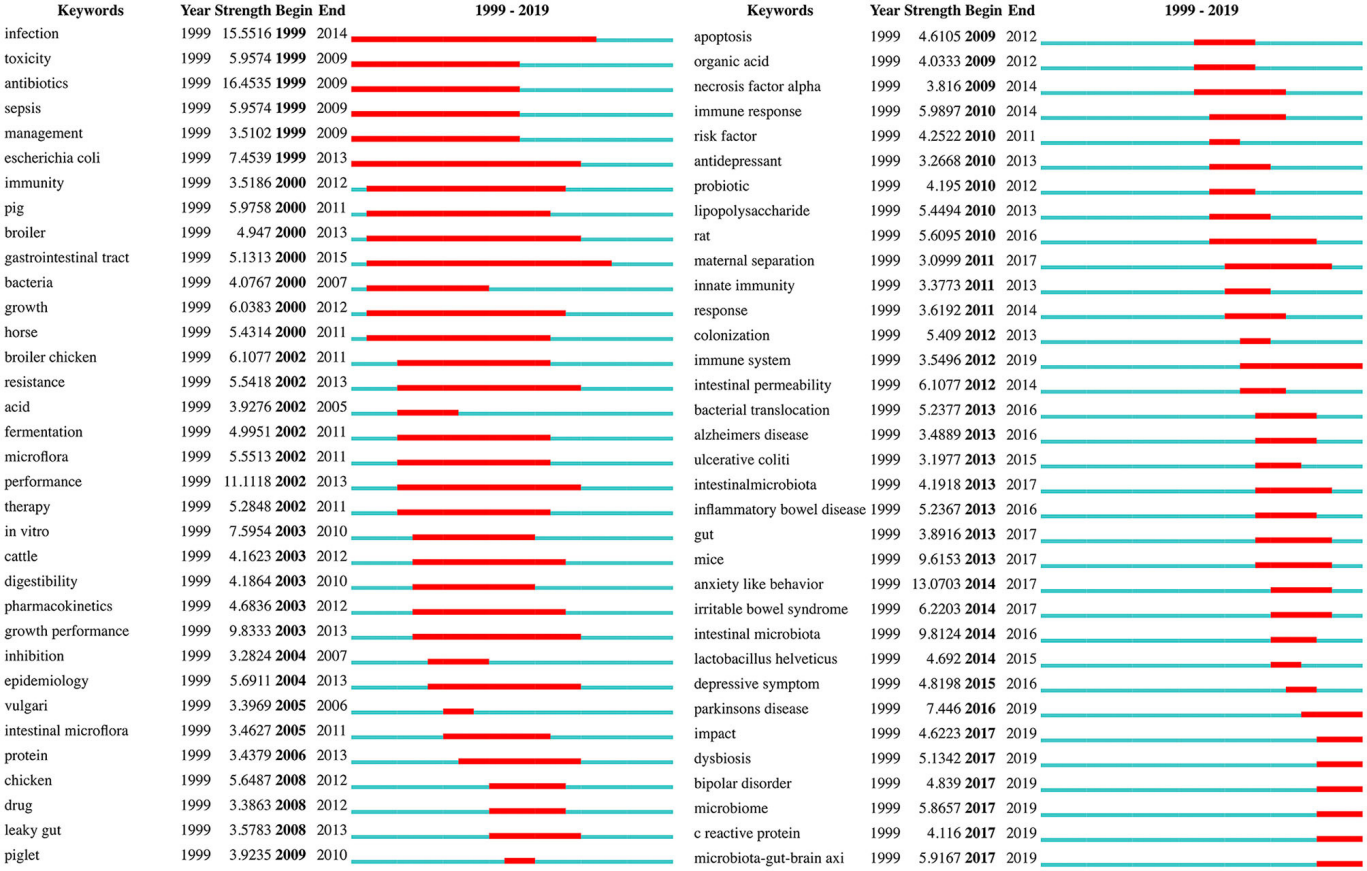
Keywords with the strongest citation bursts in publications from 1999 to 2019 on gut microbiota research in the depression field.

Burst keywords can be used to predict new frontier topics in research in a particular field. Figure 8 shows the top 68 keywords with the strongest citation bursts. “Antibiotics” was the strongest burst keyword (strength: 16.4535) in this field from 1999 to 2012, and was followed by “infection (strength: 15.5516),” “anxiety-like behavior (strength: 13.0703),” and “performance (strength: 11.1118).” The keywords with a burst lasting until 2019 included “Parkinson's disease,” “microbiota-gut-brain axis,” “microbiome,” “dysbiosis,” “bipolar disorder,” “impact,” “C reactive protein (CRP),” and “immune system,” reflecting the most recent research trends. The bursts in certain keywords can also be used to analyze the evolution of research. For instance, Figure 8 reveals that rodents such as mice and rats have replaced non-rodent animals such as piglets, cattle, and horses, as the preferred animal models in gut microbiota-depression research. The results also suggest that studies in recent decades, have increasingly focused on the relationships between the gut microbiota and potential depression-related disorders (e.g., Alzheimer's disease, inflammatory bowel disease, irritable bowel syndrome, Parkinson's disease, and bipolar disorder), as well as the underlying mechanisms, involving the immune system, intestinal permeability, bacterial translocation, cytokines such as necrosis factor-alpha, CRP, and the microbiota-gut-brain axis.

## Discussion

This is the first application of bibliometric and visual analysis methods to gut microbiota research in the depression field. A total of 1,962 publications originating from WoSCC were analyzed, and we present a comprehensive overview of the worldwide hotspots and trends in gut microbiota research in the field of depression over the past two decades. Our analysis revealed the rapid growth in the number of publications since 2010 and the close international scientific cooperation in this field. The USA, University College Cork, and John F. Cryan were the most influential country, institute, and scholar, respectively. *Brain Behavior and Immunity* and *PNAS* were the most productive and co-cited journals, respectively. The most recent research focus was on the largest theme cluster “cytokines,” thus reflecting the important research foundation in this field. Since 2013, the top two research hotspots have been “fecal microbiota” and “microbiome.” Additionally, “Parkinson's disease,” “microbiota-gut-brain axis,” “microbiome,” “dysbiosis,” “bipolar disorder,” “impact,” “CRP,” and “immune system” reflected the most recent research trends in the field. Thus, our results provided insights and valuable information regarding gut microbiota research in depression; these findings may help researchers choose suitable cooperators or journals, and promote their research through our timely review and analysis of the hotspots and research trends.

Collaboration network analysis can provide detailed information for evaluating research collaborations and identifying the key cooperators. Among countries/regions, the USA had an absolute advantage in this field, possibly because of its better economics and expenditure in scientific research; for example, a special research project on the gut microbiota-brain axis was launched in 2013 (69), thus resulting in a positive influence on research productivity and quality (70). Remarkably, China, as a developing country, has shown vast progress in this field over the past two decades. Although China was the second most productive country, its influence in this field was inferior to that of the USA because of its insufficient international cooperation. Among institutions, University College Cork was a key player, possibly because the main researchers worldwide (including John F. Cryan, Timothy G. Dinan, Gerard Clarke, and Siobhain M. O'Mahony) in this area worked at this institute, whereas the institutions and researchers in the USA were quite scattered. Overall, close international scientific cooperation was found in this field. However, we discovered that the cooperation between top researchers appears to be insufficient, a finding that may be associated with their research being comparatively full-fledged (40). For example, we found that John F. Cryan and Timothy G. Dinan, who were academic leaders at the Alimentary Pharmabiotic Centre, University College Cork, Ireland, had the most productive publications and highest citations in this field. They were the first to define the term “psychobiotic,” which refers to a live organism that produces positive effects on the mental health of people with psychiatric illness when ingested in adequate amounts (71). In addition, their research team has conducted many studies in the field of psychobiotics, funded by Science Foundation Ireland, the Health Research Board of Ireland, and the European Community's Seventh Framework Programme, in close collaboration with several companies including GlaxoSmithKline, Pfizer, Cremo, Wyeth, and Mead Johnson (26, 72).

Journal analysis and co-cited journal analysis can provide important information that can help researchers select appropriate journals for article submission. Through our research, we discovered that the top 10 most active journals published less than a quarter (10.04%) of the total publications on gut microbiota research in the depression field, thus indicating a clearly dispersed distribution of the literature distribution across journals, possibly because of the diversified research directions involving neurosciences, psychiatry, pharmacology/pharmacy, clinical neurology, and gastroenterology/hepatology. Thus, on the one hand, researchers may have many journal choices, and on the other hand, they may have difficulty in choosing the most appropriate journal because of a lack of knowledge or experience (38). In addition, there was only a 30% concordance rate between the top 10 most active journals and the top 10 co-cited journals, thus suggesting that the quality of research in this field still must be improved, and the international cooperation among researchers should be strengthened to produce high-quality research (38).

According to reference co-citation network analysis, all the top 10 highly co-cited references were located in the largest theme cluster #0, “cytokines,” which formed an important basis for studies in this field. Mounting evidence suggests that the levels of some pro-inflammatory cytokines [e.g., interleukin (IL)-1β, IL-6, IL-18, and tumor necrosis factor (TNF)-α] are increased in depressive disorder, whereas some anti-inflammatory cytokines [e.g., IL-10 and transforming growth factor (TGF)- β1] are decreased (73, 74). Some meta-analyses have provided evidence that the alterations in the levels of peripheral cytokine (e.g., IL-8, TNF-α, IL-6, and IL-10) are associated with the antidepressant treatment response in patients with major depressive disorder (MDD) (75, 76). IL-1β, IL-6, and TNF-α are the three main cytokines that play a key role in mediating signaling *via* the gut microbiome-to-brain communication pathways (77). Other cytokine signaling pathways have also been widely explored. For example, a previous study has demonstrated that live *Lactobacillus rhamnosus* (*JB-1*) can modulate the immune system by inhibiting the synthesis of TNF-induced IL-8 in the human epithelial cell lines T84 and HT-29 (78). Among the top ten co-cited references, the first was an article published by Bravo et al. in *PNAS* in 2011 (42), in which the authors further found that *L. rhamnosus* (*JB-1*) modulates the GABAergic system in mice *via* the vagus nerve, thereby exerting beneficial effects in the treatment of stress-related psychiatric disorders such as depression and anxiety. The second most co-cited reference was a review published by Cryan and Dinan in *Nature Reviews Neuroscience* in 2012 (48), in which the authors describe how the gut microbiota influences brain function and behavior through neural (vagus and enteric nervous system), endocrine (cortisol), and immune (cytokines) pathways involving bidirectional communication between the gut microbiota and the brain. Those publications have laid important research foundations in this field.

Furthermore, the dynamics of this field was partly characterized by references with citation bursts (57, 79). The reference with the strongest burst, occurring in 2010, was an article published by Desbonnet et al. in the *Journal of Psychiatric Research* in 2008 (44), in which the authors revealed that *Bifidobacteria* treatment attenuates pro-inflammatory immune responses and elevates serotonergic precursors and tryptophan, thus suggesting that depressed people, particularly those with accompanying gastrointestinal inflammation, may benefit from this probiotic, owing to its potential antidepressant properties. An article published by Maes et al. in the *Journal of Affective Disorders* in 2012 (66), had a citation burst starting in 2015 that is still ongoing. Their findings suggest that in mesenteric lymph nodes, “translocated” commensal bacteria activate immune cells and elicit IgA and IgM responses, and increased bacterial translocation may play a role in the pathophysiology of chronic depression by causing progressive amplification of inflammatory and cell-mediated immune pathways. The most recent citation bursts occurred in 2017, among which the strongest burst was due to an article published by Qin et al. in *Nature* in 2010 (64). This was the largest metagenomic survey study to date of human gut microbial genes established by metagenomic sequencing, thus setting the stage for comparing gut microbiome profiles between healthy and diseased individuals (80, 81).

According to the co-occurring keyword analysis, we identified some of the most important hotspots in this field over the past two decades, including depression, gut microbiota, anxiety, and inflammation. We found that the publication outputs have increased rapidly since 2010, partly because of the key hotspot, the second highest frequency keyword “gut microbiota,” occurring in 2011. Since 2013, “fecal microbiota” and “microbiome” have become the new top hotspots in this field. The fecal microbiota, as a proxy for the gut microbiota, have been shown to be significantly associated with depression (51, 56). Moreover, fecal microbiota transplants (FMT) technology, an ancient administration route traced back to fourth century China (82), is receiving increasing attention. In this method, stool is transferred from a healthy donor into a recipient, with the goal of replacing the recipient's “bad” bacteria with “good” bacteria to normalize the gut microbiota composition and gain therapeutic benefits (83). Accumulating clinical studies suggest that FMT has potential therapeutic effects on depression-related disorders, owing to the increase in microbiota diversity (84, 85). FMT is also called stool transplantation and is an emerging tool used in animal models in gut microbiota research. For example, Li et al. (86) have used an antibiotic cocktail to deplete the gut microbiota in mice, and then colonized the gut, *via* FMT, with the microbiota from mice exposed to chronic unpredictable mild stress. Subsequently, similar anxiety- and depression-like behavior was observed, possibly because of the significant elevation in pro-inflammatory cytokines modulated by the gut microbiota. Another hotspot is “microbiome.” The amalgam of microorganisms in a particular habitat (such as the skin, mouth, vagina, or gut) is defined as a microbiota (87). Although the term microbiome refers specifically to the collective genomes of all microorganisms in a microbiota, it is sometimes used as a synonym for microbiota (88). Microbiome-host interaction, although not a novel concept, has recently been revisited in a surge of studies, particularly those focusing on the role of the gut microbiome in regulating the maturation and functionality of the host immune system (89). Notably, the gut microbiome composition is also under the influence of multiple intrinsic and extrinsic factors, such as host genetics (90), prolonged physiological stress (91), alcohol (92), dietary habits (93), and other lifestyle factors. Additionally, an integrated analysis of gut microbial community profiling and the host metabolomic signatures can help to provide a comprehensive understanding of microbiome-host interaction, thus providing a basis for studying the pathogenesis of depression (94). Notably, the traditional concepts will probably need to be revised by considering the reciprocal interactions between the gut microbiome and xenobiotics, termed the “microbiome-xenobiotic interactions” (95). For example, a recent study has shown that administration of psychotropics (such as fluoxetine, lithium, valproate, and aripiprazole) significantly alters the gut microbiome composition (96). Despite the increasing recognition of these hotspots, as these research directions develop, further innovations and breakthroughs may be hindered. Therefore, there is a need to pay more attention to frontier Research Topics identified by the burst keyword analysis in the years ahead.

Of note, many strong burst keywords associated with emerging areas of gut microbiota-depression-related research have appeared in recent years, including anxiety-like behavior, intestinal microbiota, and mice. Moreover, some keywords, such as the abovementioned hotspot “microbiome,” had bursts that are currently ongoing, among which the top keyword was “Parkinson's disease.”

Parkinson's disease is the second most common neurodegenerative disorder after Alzheimer's disease, with a median incidence in developed countries of 14 per 100,000 people in the total population, and 160 per 100,000 people at least 65 years of age (97). Apart from classical motor symptoms such as tremors, bradykinesia, and rigidity, some non-motor symptoms, including depression, anxiety, apathy, and psychosis, also affect quality of life in people with Parkinson's disease (98). Depression occurs in approximately 35% of people with Parkinson's disease, and the alterations in neurotransmitter systems, as well as neurotrophic factors, neuropeptides, immunomodulatory mediators, and stress hormones, may be associated with the depression observed in these individuals (99). Previous evidence supports the antidepressant properties of minocycline (a broad-spectrum tetracycline antibiotic), owing to its suppression of microglial activation, and neuro-protective or anti-inflammatory actions (100, 101). A population-based cohort study in older people (HEllenic Longitudinal Investigation of Aging and Diet) has found that adherence to the Mediterranean diet is associated with a lower probability of non-motor markers of prodromal Parkinson's disease, mainly depression and constipation (102). This finding may be due to decreased matrix metalloproteinase 9 activity (103), thereby maintaining the intestinal microbiota diversity and decreasing the severity of depression (104, 105). All the above findings suggest that manipulation of the microbiota may be beneficial in alleviating mood disorders in Parkinson's disease (104). Thus, modifying the microbiota to modulate depression in Parkinson's disease may be a future research direction. Moreover, depression, as a prodromal biomarker, can be used to identify the risk of developing Parkinson's disease, and assessment of the gut microbiota may also be considered an early biomarker of this disease in the future (104).

Other frontier topics in this field should also be noted, such as “microbiota-gut-brain axis,” “dysbiosis,” “CRP,” and “immune system,” which have currently ongoing bursts. Depression is a form of mood disorders that probably results from immunological deregulation (89). The immune system (including both the innate and adaptive immune system) is an important part of the microbiota-gut-brain axis that mediates the communication between the gut microbiota and CNS (106). The communication between the brain and the immune system occurs through different pathways, including neural and humoral pathways (107). The gut microbiota can activate epithelial cell gene expression *via* molecules such as Toll-like receptor (TLR), an innate immune system protein (108), thus inducing the production and release of pro-inflammatory cytokines (107). These cytokines then cross the blood brain barrier (BBB), possibly *via* a volume diffusion mechanism (109). Increased gut dysbiosis can also increase mucosal barrier permeability, thereby allowing bacteria and their pro-inflammatory products into the circulatory system and contributing to a chronic pro-inflammatory state (110). In individuals with MDD compared with healthy controls, higher elevated peripheral levels of CRP, an immune-inflammatory marker, have been confirmed by meta-analysis (111). CRP has also been demonstrated to have a relationship with depressive symptoms in obese people (112, 113). The probable pathway linking inflammation to these symptoms is an obesity-depression-inflammation cycle, involving systemic low-grade inflammation, partly owing to the changes in the gut microbiota (113). Thus, future clinical studies should focus on the feasibility of using anti-inflammatory strategies for patients with obesity and MDD, who might possibly benefit from reducing systemic inflammatory biomarkers such as CRP.

Our study has several limitations. First, publications were retrieved from only the WoSCC database, which may have led to bias and incompleteness in the included studies. In addition, our retrieval strategy may not have identified all the relevant references, owing to the limited terms, types of literature, and language; thus, our findings may not be comprehensive. Second, we cannot ensure that each publication retrieved was completely relevant to the topic meeting the search criteria. Third, some authors have the same name, and some keywords have different expressions, for example, “gut-brain axis” and “brain-gut axis”; thus, bias may have still existed despite our normalization procedures. Future studies should consider more databases, such as Scopus, more accurate and comprehensive search terms, and the latest software versions, in which bugs are addressed. However, we believe that our findings reflect the overall state of, and general trends in, this field, because the number of publications in our analysis was sufficiently large. We also believe that these limitations may be noted in future similar studies and addressed wherever possible.

## Conclusions

This study comprised a bibliometric and visual analysis of gut microbiota research in the field of depression over the past two decades. The number of publications has been rapidly growing since 2010. Overall, close international scientific cooperation was observed in this field, but more cooperation among researchers may be necessary. There were multiple journals for researchers to choose from because of the diversified research directions. The most recent research focus was on “cytokines,” thus reflecting the important research foundation in this field. “Fecal microbiota” and “microbiome” have become the top two research hotspots since 2013. However, “Parkinson's disease,” “microbiota-gut-brain axis,” “microbiome,” “dysbiosis,” “bipolar disorder,” “impact,” “CRP,” and “immune system” were identified as new frontiers of research, whose bursts are currently ongoing. Therefore, our timely review and analysis of the hotspots and research trends may promote the development of this field.

## Data Availability Statement

The raw data supporting the conclusions of this article will be made available by the authors, without undue reservation.

## Author Contributions

DS and YW conceived and designed the study. XZ wrote the original draft preparation. HL assisted in literature searching. JH conducted analysis based on WoS. XN, LL, and MZ conducted the CiteSpace analysis. SH, HC, and TX provided the figures and tablets. CQ, ZW, SD, and YT reviewed and edited the manuscript for content and style. All authors contributed to the article and approved the submitted version.

## Conflict of Interest

The authors declare that the research was conducted in the absence of any commercial or financial relationships that could be construed as a potential conflict of interest.
